# Supplementary material for: Pyruvate Dehydrogenase Kinase 1 inhibition mediated oxidative phosphorylation enhancement in cartilage promotes osteoarthritis progression
Source: BMC Musculoskelet Disord. 2023 Jul 20;24:597. doi: 10.1186/s12891-023-06585-6 (PMC10357736; doi:10.1186/s12891-023-06585-6)
Supplement: Supplementary file 1 — Additional file 1. [file 12891_2023_6585_MOESM1_ESM.zip › BP.Gsea.1653623667859/GOBP_CYTOPLASMIC_TRANSLATION.html]

Details for gene set GOBP\_CYTOPLASMIC\_TRANSLATION[GSEA]

|  || Dataset | 55457\_collapsed\_to\_symbols.cls.cls #OA\_versus\_normal.cls.cls #OA\_versus\_normal\_repos |
| Phenotype | cls.cls#OA\_versus\_normal\_repos |
| Upregulated in class | normal |
| GeneSet | GOBP\_CYTOPLASMIC\_TRANSLATION |
| Enrichment Score (ES) | -0.54504925 |
| Normalized Enrichment Score (NES) | -2.435192 |
| Nominal p-value | 0.0 |
| FDR q-value | 0.0 |
| FWER p-Value | 0.0 |
Table: GSEA Results Summary

  

Fig 1: Enrichment plot: GOBP\_CYTOPLASMIC\_TRANSLATION      
 Profile of the Running ES Score & Positions of GeneSet Members on the Rank Ordered List

  

| SYMBOL | TITLE | RANK IN GENE LIST | RANK METRIC SCORE | RUNNING ES | CORE ENRICHMENT || 1 | GSPT2 | G1 to S phase transition 2 [Source:HGNC Symbol;Acc:HGNC:4622] | 42 | 1.008 | 0.0159 | No |
| 2 | NCK1 | NCK adaptor protein 1 [Source:HGNC Symbol;Acc:HGNC:7664] | 222 | 0.804 | 0.0172 | No |
| 3 | MCTS1 | MCTS1 re-initiation and release factor [Source:HGNC Symbol;Acc:HGNC:23357] | 527 | 0.649 | 0.0058 | No |
| 4 | EIF3I | eukaryotic translation initiation factor 3 subunit I [Source:HGNC Symbol;Acc:HGNC:3272] | 697 | 0.594 | 0.0039 | No |
| 5 | DPH5 | diphthamide biosynthesis 5 [Source:HGNC Symbol;Acc:HGNC:24270] | 774 | 0.575 | 0.0089 | No |
| 6 | DHX29 | DExH-box helicase 29 [Source:HGNC Symbol;Acc:HGNC:15815] | 1012 | 0.515 | 0.0001 | No |
| 7 | EIF2D | eukaryotic translation initiation factor 2D [Source:HGNC Symbol;Acc:HGNC:6583] | 1162 | 0.485 | -0.0023 | No |
| 8 | DRG1 | developmentally regulated GTP binding protein 1 [Source:HGNC Symbol;Acc:HGNC:3029] | 1358 | 0.449 | -0.0090 | No |
| 9 | RBM4 | RNA binding motif protein 4 [Source:HGNC Symbol;Acc:HGNC:9901] | 1970 | 0.364 | -0.0499 | No |
| 10 | FTSJ1 | FtsJ RNA 2'-O-methyltransferase 1 [Source:HGNC Symbol;Acc:HGNC:13254] | 2664 | 0.284 | -0.0988 | No |
| 11 | LIN28A | lin-28 homolog A [Source:HGNC Symbol;Acc:HGNC:15986] | 3140 | 0.236 | -0.1315 | No |
| 12 | RPL36A | ribosomal protein L36a [Source:HGNC Symbol;Acc:HGNC:10359] | 3387 | 0.213 | -0.1467 | No |
| 13 | EIF2B3 | eukaryotic translation initiation factor 2B subunit gamma [Source:HGNC Symbol;Acc:HGNC:3259] | 3461 | 0.206 | -0.1485 | No |
| 14 | SLBP | stem-loop binding protein [Source:HGNC Symbol;Acc:HGNC:10904] | 3703 | 0.185 | -0.1638 | No |
| 15 | RPL18 | ribosomal protein L18 [Source:HGNC Symbol;Acc:HGNC:10310] | 3749 | 0.182 | -0.1639 | No |
| 16 | PKM | pyruvate kinase M1/2 [Source:HGNC Symbol;Acc:HGNC:9021] | 3963 | 0.159 | -0.1775 | No |
| 17 | EIF3D | eukaryotic translation initiation factor 3 subunit D [Source:HGNC Symbol;Acc:HGNC:3278] | 4032 | 0.153 | -0.1799 | No |
| 18 | RPL18A | ribosomal protein L18a [Source:HGNC Symbol;Acc:HGNC:10311] | 4049 | 0.151 | -0.1783 | No |
| 19 | RPL26L1 | ribosomal protein L26 like 1 [Source:HGNC Symbol;Acc:HGNC:17050] | 4584 | 0.106 | -0.2181 | No |
| 20 | DNAJC24 | DnaJ heat shock protein family (Hsp40) member C24 [Source:HGNC Symbol;Acc:HGNC:26979] | 4599 | 0.105 | -0.2172 | No |
| 21 | HNRNPD | heterogeneous nuclear ribonucleoprotein D [Source:HGNC Symbol;Acc:HGNC:5036] | 4830 | 0.085 | -0.2336 | No |
| 22 | EIF2S2 | eukaryotic translation initiation factor 2 subunit beta [Source:HGNC Symbol;Acc:HGNC:3266] | 4838 | 0.084 | -0.2325 | No |
| 23 | PAIP1 | poly(A) binding protein interacting protein 1 [Source:HGNC Symbol;Acc:HGNC:16945] | 4851 | 0.083 | -0.2319 | No |
| 24 | RPL10A | ribosomal protein L10a [Source:HGNC Symbol;Acc:HGNC:10299] | 4974 | 0.074 | -0.2400 | No |
| 25 | UBA52 | ubiquitin A-52 residue ribosomal protein fusion product 1 [Source:HGNC Symbol;Acc:HGNC:12458] | 4978 | 0.074 | -0.2389 | No |
| 26 | EIF4H | eukaryotic translation initiation factor 4H [Source:HGNC Symbol;Acc:HGNC:12741] | 5118 | 0.062 | -0.2485 | No |
| 27 | RPS26 | ribosomal protein S26 [Source:HGNC Symbol;Acc:HGNC:10414] | 5368 | 0.043 | -0.2672 | No |
| 28 | AARS1 | alanyl-tRNA synthetase 1 [Source:HGNC Symbol;Acc:HGNC:20] | 5703 | 0.015 | -0.2931 | No |
| 29 | DPH1 | diphthamide biosynthesis 1 [Source:HGNC Symbol;Acc:HGNC:3003] | 5835 | 0.006 | -0.3032 | No |
| 30 | RPS19 | ribosomal protein S19 [Source:HGNC Symbol;Acc:HGNC:10402] | 5952 | -0.002 | -0.3123 | No |
| 31 | RPL15 | ribosomal protein L15 [Source:HGNC Symbol;Acc:HGNC:10306] | 6185 | -0.019 | -0.3301 | No |
| 32 | RPL35A | ribosomal protein L35a [Source:HGNC Symbol;Acc:HGNC:10345] | 6383 | -0.034 | -0.3449 | No |
| 33 | CPEB1 | cytoplasmic polyadenylation element binding protein 1 [Source:HGNC Symbol;Acc:HGNC:21744] | 6435 | -0.038 | -0.3481 | No |
| 34 | RPL8 | ribosomal protein L8 [Source:HGNC Symbol;Acc:HGNC:10368] | 6606 | -0.050 | -0.3605 | No |
| 35 | DENR | density regulated re-initiation and release factor [Source:HGNC Symbol;Acc:HGNC:2769] | 6611 | -0.050 | -0.3598 | No |
| 36 | METTL3 | "methyltransferase 3, N6-adenosine-methyltransferase complex catalytic subunit [Source:HGNC Symbol;Acc:HGNC:17563]" | 6685 | -0.055 | -0.3645 | No |
| 37 | DRG2 | developmentally regulated GTP binding protein 2 [Source:HGNC Symbol;Acc:HGNC:3030] | 6690 | -0.055 | -0.3638 | No |
| 38 | RPL12 | ribosomal protein L12 [Source:HGNC Symbol;Acc:HGNC:10302] | 6845 | -0.067 | -0.3746 | No |
| 39 | RPL36 | ribosomal protein L36 [Source:HGNC Symbol;Acc:HGNC:13631] | 6860 | -0.068 | -0.3744 | No |
| 40 | EIF2S3 | eukaryotic translation initiation factor 2 subunit gamma [Source:HGNC Symbol;Acc:HGNC:3267] | 6870 | -0.069 | -0.3738 | No |
| 41 | EIF5 | eukaryotic translation initiation factor 5 [Source:HGNC Symbol;Acc:HGNC:3299] | 6934 | -0.074 | -0.3773 | No |
| 42 | RPS27A | ribosomal protein S27a [Source:HGNC Symbol;Acc:HGNC:10417] | 6973 | -0.078 | -0.3788 | No |
| 43 | RPL35 | ribosomal protein L35 [Source:HGNC Symbol;Acc:HGNC:10344] | 7563 | -0.124 | -0.4225 | No |
| 44 | FMR1 | FMRP translational regulator 1 [Source:HGNC Symbol;Acc:HGNC:3775] | 7944 | -0.151 | -0.4494 | No |
| 45 | EIF4B | eukaryotic translation initiation factor 4B [Source:HGNC Symbol;Acc:HGNC:3285] | 8051 | -0.159 | -0.4547 | No |
| 46 | CPEB3 | cytoplasmic polyadenylation element binding protein 3 [Source:HGNC Symbol;Acc:HGNC:21746] | 8060 | -0.160 | -0.4522 | No |
| 47 | RPS21 | ribosomal protein S21 [Source:HGNC Symbol;Acc:HGNC:10409] | 8394 | -0.185 | -0.4748 | No |
| 48 | RPS9 | ribosomal protein S9 [Source:HGNC Symbol;Acc:HGNC:10442] | 8508 | -0.194 | -0.4799 | No |
| 49 | RPL29 | ribosomal protein L29 [Source:HGNC Symbol;Acc:HGNC:10331] | 8661 | -0.205 | -0.4879 | No |
| 50 | RPSA | ribosomal protein SA [Source:HGNC Symbol;Acc:HGNC:6502] | 8866 | -0.222 | -0.4997 | No |
| 51 | RPL28 | ribosomal protein L28 [Source:HGNC Symbol;Acc:HGNC:10330] | 8985 | -0.230 | -0.5045 | No |
| 52 | RPLP2 | ribosomal protein lateral stalk subunit P2 [Source:HGNC Symbol;Acc:HGNC:10377] | 9185 | -0.246 | -0.5154 | No |
| 53 | EIF4A2 | eukaryotic translation initiation factor 4A2 [Source:HGNC Symbol;Acc:HGNC:3284] | 9251 | -0.252 | -0.5157 | No |
| 54 | RPL37 | ribosomal protein L37 [Source:HGNC Symbol;Acc:HGNC:10347] | 9265 | -0.253 | -0.5119 | No |
| 55 | RPS25 | ribosomal protein S25 [Source:HGNC Symbol;Acc:HGNC:10413] | 9282 | -0.254 | -0.5083 | No |
| 56 | YBX1 | Y-box binding protein 1 [Source:HGNC Symbol;Acc:HGNC:8014] | 9623 | -0.285 | -0.5295 | No |
| 57 | DPH2 | diphthamide biosynthesis 2 [Source:HGNC Symbol;Acc:HGNC:3004] | 9661 | -0.288 | -0.5269 | No |
| 58 | RPL4 | ribosomal protein L4 [Source:HGNC Symbol;Acc:HGNC:10353] | 9770 | -0.298 | -0.5297 | No |
| 59 | HNRNPU | heterogeneous nuclear ribonucleoprotein U [Source:HGNC Symbol;Acc:HGNC:5048] | 9847 | -0.306 | -0.5298 | No |
| 60 | EIF3M | eukaryotic translation initiation factor 3 subunit M [Source:HGNC Symbol;Acc:HGNC:24460] | 9915 | -0.313 | -0.5291 | No |
| 61 | RWDD1 | RWD domain containing 1 [Source:HGNC Symbol;Acc:HGNC:20993] | 10087 | -0.330 | -0.5362 | No |
| 62 | RPL5 | ribosomal protein L5 [Source:HGNC Symbol;Acc:HGNC:10360] | 10201 | -0.341 | -0.5386 | Yes |
| 63 | ETF1 | eukaryotic translation termination factor 1 [Source:HGNC Symbol;Acc:HGNC:3477] | 10208 | -0.341 | -0.5325 | Yes |
| 64 | RPL24 | ribosomal protein L24 [Source:HGNC Symbol;Acc:HGNC:10325] | 10263 | -0.347 | -0.5301 | Yes |
| 65 | RPL22 | ribosomal protein L22 [Source:HGNC Symbol;Acc:HGNC:10315] | 10270 | -0.348 | -0.5240 | Yes |
| 66 | RPS8 | ribosomal protein S8 [Source:HGNC Symbol;Acc:HGNC:10441] | 10525 | -0.376 | -0.5367 | Yes |
| 67 | RPL31 | ribosomal protein L31 [Source:HGNC Symbol;Acc:HGNC:10334] | 10533 | -0.377 | -0.5301 | Yes |
| 68 | RPS14 | ribosomal protein S14 [Source:HGNC Symbol;Acc:HGNC:10387] | 10558 | -0.379 | -0.5247 | Yes |
| 69 | CSDE1 | cold shock domain containing E1 [Source:HGNC Symbol;Acc:HGNC:29905] | 10559 | -0.379 | -0.5175 | Yes |
| 70 | RPS7 | ribosomal protein S7 [Source:HGNC Symbol;Acc:HGNC:10440] | 10608 | -0.384 | -0.5139 | Yes |
| 71 | DHX9 | DExH-box helicase 9 [Source:HGNC Symbol;Acc:HGNC:2750] | 10627 | -0.386 | -0.5080 | Yes |
| 72 | RPL10 | ribosomal protein L10 [Source:HGNC Symbol;Acc:HGNC:10298] | 10684 | -0.391 | -0.5049 | Yes |
| 73 | CNBP | CCHC-type zinc finger nucleic acid binding protein [Source:HGNC Symbol;Acc:HGNC:13164] | 10707 | -0.394 | -0.4992 | Yes |
| 74 | RPS11 | ribosomal protein S11 [Source:HGNC Symbol;Acc:HGNC:10384] | 10734 | -0.397 | -0.4936 | Yes |
| 75 | RPL17 | ribosomal protein L17 [Source:HGNC Symbol;Acc:HGNC:10307] | 10806 | -0.406 | -0.4915 | Yes |
| 76 | RPL7A | ribosomal protein L7a [Source:HGNC Symbol;Acc:HGNC:10364] | 10811 | -0.406 | -0.4840 | Yes |
| 77 | RPS4X | ribosomal protein S4 X-linked [Source:HGNC Symbol;Acc:HGNC:10424] | 10812 | -0.406 | -0.4763 | Yes |
| 78 | RPS29 | ribosomal protein S29 [Source:HGNC Symbol;Acc:HGNC:10419] | 10913 | -0.419 | -0.4761 | Yes |
| 79 | RPL13 | ribosomal protein L13 [Source:HGNC Symbol;Acc:HGNC:10303] | 11013 | -0.431 | -0.4757 | Yes |
| 80 | GSPT1 | G1 to S phase transition 1 [Source:HGNC Symbol;Acc:HGNC:4621] | 11070 | -0.441 | -0.4717 | Yes |
| 81 | RPL38 | ribosomal protein L38 [Source:HGNC Symbol;Acc:HGNC:10349] | 11122 | -0.446 | -0.4672 | Yes |
| 82 | RPL6 | ribosomal protein L6 [Source:HGNC Symbol;Acc:HGNC:10362] | 11129 | -0.447 | -0.4591 | Yes |
| 83 | RPL14 | ribosomal protein L14 [Source:HGNC Symbol;Acc:HGNC:10305] | 11154 | -0.451 | -0.4524 | Yes |
| 84 | SYNCRIP | synaptotagmin binding cytoplasmic RNA interacting protein [Source:HGNC Symbol;Acc:HGNC:16918] | 11182 | -0.454 | -0.4459 | Yes |
| 85 | RPS5 | ribosomal protein S5 [Source:HGNC Symbol;Acc:HGNC:10426] | 11206 | -0.458 | -0.4389 | Yes |
| 86 | ZC3H15 | zinc finger CCCH-type containing 15 [Source:HGNC Symbol;Acc:HGNC:29528] | 11243 | -0.463 | -0.4329 | Yes |
| 87 | RPS3 | ribosomal protein S3 [Source:HGNC Symbol;Acc:HGNC:10420] | 11245 | -0.463 | -0.4242 | Yes |
| 88 | RPL11 | ribosomal protein L11 [Source:HGNC Symbol;Acc:HGNC:10301] | 11252 | -0.464 | -0.4158 | Yes |
| 89 | RPS10 | ribosomal protein S10 [Source:HGNC Symbol;Acc:HGNC:10383] | 11408 | -0.488 | -0.4187 | Yes |
| 90 | RPL32 | ribosomal protein L32 [Source:HGNC Symbol;Acc:HGNC:10336] | 11447 | -0.496 | -0.4122 | Yes |
| 91 | RPS20 | ribosomal protein S20 [Source:HGNC Symbol;Acc:HGNC:10405] | 11507 | -0.502 | -0.4073 | Yes |
| 92 | RPL21 | ribosomal protein L21 [Source:HGNC Symbol;Acc:HGNC:10313] | 11647 | -0.524 | -0.4082 | Yes |
| 93 | RPS6 | ribosomal protein S6 [Source:HGNC Symbol;Acc:HGNC:10429] | 11662 | -0.526 | -0.3992 | Yes |
| 94 | RPS15 | ribosomal protein S15 [Source:HGNC Symbol;Acc:HGNC:10388] | 11671 | -0.527 | -0.3898 | Yes |
| 95 | RPS16 | ribosomal protein S16 [Source:HGNC Symbol;Acc:HGNC:10396] | 11755 | -0.541 | -0.3860 | Yes |
| 96 | RPL27 | ribosomal protein L27 [Source:HGNC Symbol;Acc:HGNC:10328] | 11765 | -0.544 | -0.3763 | Yes |
| 97 | RPL19 | ribosomal protein L19 [Source:HGNC Symbol;Acc:HGNC:10312] | 11967 | -0.587 | -0.3809 | Yes |
| 98 | YBX3 | Y-box binding protein 3 [Source:HGNC Symbol;Acc:HGNC:2428] | 12024 | -0.599 | -0.3739 | Yes |
| 99 | MTOR | mechanistic target of rapamycin kinase [Source:HGNC Symbol;Acc:HGNC:3942] | 12047 | -0.605 | -0.3641 | Yes |
| 100 | RPS27 | ribosomal protein S27 [Source:HGNC Symbol;Acc:HGNC:10416] | 12071 | -0.612 | -0.3542 | Yes |
| 101 | EIF4A1 | eukaryotic translation initiation factor 4A1 [Source:HGNC Symbol;Acc:HGNC:3282] | 12111 | -0.623 | -0.3454 | Yes |
| 102 | EIF4G1 | eukaryotic translation initiation factor 4 gamma 1 [Source:HGNC Symbol;Acc:HGNC:3296] | 12122 | -0.625 | -0.3343 | Yes |
| 103 | RPS17 | ribosomal protein S17 [Source:HGNC Symbol;Acc:HGNC:10397] | 12130 | -0.626 | -0.3229 | Yes |
| 104 | RPL30 | ribosomal protein L30 [Source:HGNC Symbol;Acc:HGNC:10333] | 12140 | -0.629 | -0.3116 | Yes |
| 105 | RPS12 | ribosomal protein S12 [Source:HGNC Symbol;Acc:HGNC:10385] | 12156 | -0.632 | -0.3008 | Yes |
| 106 | RPL13A | ribosomal protein L13a [Source:HGNC Symbol;Acc:HGNC:10304] | 12200 | -0.645 | -0.2918 | Yes |
| 107 | RPS24 | ribosomal protein S24 [Source:HGNC Symbol;Acc:HGNC:10411] | 12223 | -0.652 | -0.2811 | Yes |
| 108 | RPLP0 | ribosomal protein lateral stalk subunit P0 [Source:HGNC Symbol;Acc:HGNC:10371] | 12285 | -0.672 | -0.2731 | Yes |
| 109 | RPS15A | ribosomal protein S15a [Source:HGNC Symbol;Acc:HGNC:10389] | 12291 | -0.674 | -0.2607 | Yes |
| 110 | RPS13 | ribosomal protein S13 [Source:HGNC Symbol;Acc:HGNC:10386] | 12334 | -0.687 | -0.2509 | Yes |
| 111 | RPL3 | ribosomal protein L3 [Source:HGNC Symbol;Acc:HGNC:10332] | 12350 | -0.691 | -0.2389 | Yes |
| 112 | EIF3A | eukaryotic translation initiation factor 3 subunit A [Source:HGNC Symbol;Acc:HGNC:3271] | 12402 | -0.707 | -0.2294 | Yes |
| 113 | RPLP1 | ribosomal protein lateral stalk subunit P1 [Source:HGNC Symbol;Acc:HGNC:10372] | 12467 | -0.734 | -0.2204 | Yes |
| 114 | RPL7 | ribosomal protein L7 [Source:HGNC Symbol;Acc:HGNC:10363] | 12519 | -0.757 | -0.2100 | Yes |
| 115 | RPS28 | ribosomal protein S28 [Source:HGNC Symbol;Acc:HGNC:10418] | 12526 | -0.761 | -0.1960 | Yes |
| 116 | NEMF | nuclear export mediator factor [Source:HGNC Symbol;Acc:HGNC:10663] | 12531 | -0.762 | -0.1818 | Yes |
| 117 | RPS2 | ribosomal protein S2 [Source:HGNC Symbol;Acc:HGNC:10404] | 12541 | -0.769 | -0.1678 | Yes |
| 118 | RPL23 | ribosomal protein L23 [Source:HGNC Symbol;Acc:HGNC:10316] | 12558 | -0.777 | -0.1543 | Yes |
| 119 | PABPC1 | poly(A) binding protein cytoplasmic 1 [Source:HGNC Symbol;Acc:HGNC:8554] | 12562 | -0.778 | -0.1397 | Yes |
| 120 | RPL27A | ribosomal protein L27a [Source:HGNC Symbol;Acc:HGNC:10329] | 12592 | -0.792 | -0.1269 | Yes |
| 121 | RPL34 | ribosomal protein L34 [Source:HGNC Symbol;Acc:HGNC:10340] | 12602 | -0.799 | -0.1124 | Yes |
| 122 | RPS18 | ribosomal protein S18 [Source:HGNC Symbol;Acc:HGNC:10401] | 12611 | -0.804 | -0.0977 | Yes |
| 123 | RPS23 | ribosomal protein S23 [Source:HGNC Symbol;Acc:HGNC:10410] | 12661 | -0.834 | -0.0856 | Yes |
| 124 | RPL41 | ribosomal protein L41 [Source:HGNC Symbol;Acc:HGNC:10354] | 12674 | -0.847 | -0.0704 | Yes |
| 125 | YTHDF2 | YTH N6-methyladenosine RNA binding protein 2 [Source:HGNC Symbol;Acc:HGNC:31675] | 12694 | -0.860 | -0.0555 | Yes |
| 126 | RPL37A | ribosomal protein L37a [Source:HGNC Symbol;Acc:HGNC:10348] | 12754 | -0.918 | -0.0427 | Yes |
| 127 | RPS3A | ribosomal protein S3A [Source:HGNC Symbol;Acc:HGNC:10421] | 12758 | -0.921 | -0.0254 | Yes |
| 128 | RPL23A | ribosomal protein L23a [Source:HGNC Symbol;Acc:HGNC:10317] | 12785 | -0.944 | -0.0094 | Yes |
| 129 | RPL39 | ribosomal protein L39 [Source:HGNC Symbol;Acc:HGNC:10350] | 12797 | -0.963 | 0.0081 | Yes |
Table: GSEA details [plain text format]

  

Fig 2: GOBP\_CYTOPLASMIC\_TRANSLATION      
 Blue-Pink O' Gram in the Space of the Analyzed GeneSet

  

Fig 3: GOBP\_CYTOPLASMIC\_TRANSLATION: Random ES distribution      
 Gene set null distribution of ES for **GOBP\_CYTOPLASMIC\_TRANSLATION**
